# Effects of therapeutic and aerobic exercise programs in temporomandibular disorder-associated headaches

**DOI:** 10.1590/1678-7757-2021-0059

**Published:** 2021-09-08

**Authors:** Paula Manuela Mendes MOLEIRINHO-ALVES, André Mariz Coelho Santos de ALMEIDA, Pedro Miguel Teixeira Carvas CEBOLA, Raul Alexandre Nunes da Silva OLIVEIRA, Pedro Luís Camecelha de PEZARAT-CORREIA

**Affiliations:** 1 Universidade de Lisboa Faculdade de Motricidade Humana CIPER Laboratório de Função Neuromuscular Lisboa Portugal Universidade de Lisboa, Faculdade de Motricidade Humana, CIPER Laboratório de Função Neuromuscular, Lisboa, Portugal; Centro de Investigação Interdisciplinar Egas Moniz, Escola Superior de Saúde Egas Moniz, Monte de Caparica, Portugal; Cuf Tejo Hospital, Lisboa, Portugal;; Centro de Investigação Interdisciplinar Egas Moniz Escola Superior de Saúde Egas Moniz Monte de Caparica Portugal; Cuf Tejo Hospital Lisboa Portugal; 2 Instituto Universitário Egas Moniz Centro de Investigação Interdisciplinar Egas Moniz Monte de Caparica Portugal Instituto Universitário Egas Moniz, Centro de Investigação Interdisciplinar Egas Moniz; Monte de Caparica, Portugal; Cuf Tejo Hospital, Lisboa, Portugal;; Cuf Tejo Hospital Lisboa Portugal; 3 Universidade de Lisboa Faculdade de Motricidade Humana CIPER Laboratório de Função Neuromuscular Lisboa Portugal UUniversidade de Lisboa, Faculdade de Motricidade Humana, CIPER Laboratório de Função Neuromuscular Lisboa, Portugal;

**Keywords:** Temporomandibular Joint Disorders, Headache, HIT-6

## Abstract

**Objective:**

To assess the effects of three 8-week exercise programs on the frequency, intensity, and impact of headaches in patients with headache attributed to temporomandibular disorder (TMD).

**Methodology:**

Thirty-six patients diagnosed with headache attributed to TMD participated in the study and were divided into three groups of 12 patients: a therapeutic exercise program (G1, mean age: 26.3±5.6 years), a therapeutic and aerobic exercise program (G2, mean age: 26.0±4.6 years), and an aerobic exercise program (G3, 25.8±2.94 years). Headache frequency and intensity were evaluated using a headache diary, and the adverse headache impact was evaluated using the Headache Impact Test (HIT-6). The intensity was reported using the numerical pain rating scale. These parameters were evaluated twice at baseline (A01/A02), at the end of the 8-week intervention period (A1), and 8–12 weeks after the end of the intervention (A2).

**Results:**

At A1, none of the G2 patients reported having headaches, in G1, only two patients reported headaches, and in G3, ten patients reported headache. The headache intensity scores (0.3 [95% CI: -0.401, 1.068]), (0.0 [95% CI: -0.734, 0.734]) and HIT-6 (50.7 [95% CI: 38.008, 63.459]), (49.5 [95% CI: 36.808, 62.259]), significantly decreased in G1 and G2 at A1. At A2 headache intensity scores (0.5 [95% CI: -0.256, 1.256]), (0.0 [95% CI: -0.756, 0.756]) and HIT-6 (55.1 [95% CI: 42.998, 67.268]), (51.7 [95% CI: 39.532, 63.802]) in G1 and G2 haven’t change significantly. The effects obtained immediately after the completion of the intervention programs were maintained until the final follow-up in all groups.

**Conclusion:**

The programs conducted by G1 (therapeutic exercises) and G2 (therapeutic and aerobic exercise) had significant results at A1 and A2.

## Introduction

Headaches and temporomandibular disorders (TMDs) frequently occur simultaneously,^1^ and associated to each other.^2,3^ Both are comorbid conditions, that is, the presence of one increases the frequency of the other^4^; for example, the more TMD symptoms a person experiences, the more frequent their headaches are, and vice versa.^5^ Due to the biomechanical aspects of TMD and the headaches, there is a constant interaction between these diseases, since TMD may lead to the development of headaches, due to the pain in the masticatory muscles. This disorder can also become a predisposing and aggravating factor for the onset of headaches.^6^ The overlap between TMD symptoms and headaches hinders the process of diagnosis and treatment. Moreover, the high level of association between the diagnostic criteria and the peripheral and central nervous structures involved in the two disorders further complicates the process.^1,7^

Physiotherapy is the most frequently used alternative or complementary headache intervention strategy.^8^ Therapeutic exercises are the most common interventions,^9^ including joint mobilization, therapeutic massage and other body-based therapies; however, evidence supporting the effectiveness of these interventions is still scarce.^10,11^ Therefore, further investigations should evaluate the effects of physical therapy individually as a part of a multimodal approach,^11^ since studies present multiple types of interventions, hindering results comparisons.^10,12^ Aerobic exercise seems to be effective in decreasing the intensity and frequency of headaches, but the heterogeneity of study protocols does not allow us to determine the intensity of aerobic exercise needed and the most appropriate modality to reduce symptoms; therefore, an exercise prescription cannot often be formulated.^13^ To date, evidence on the combined effect of therapeutic and aerobic exercise are scarce. The null hypothesis is that patients with headache attributed to TMD undergoing a combined program of therapeutic exercises with aerobic exercises (G2) will not show a significant reduction of the frequency, intensity and adverse impact of headaches. Thus, this study aimed to evaluate the effects of three different 8-week intervention programs (therapeutic, therapeutic with aerobic exercise, and aerobic) on the frequency and intensity of headaches and the adverse headaches impact on patients with muscle TMD.

## Methods

A non-probabilistic convenience sampling method was used, in which patients were recruited at the Egas Moniz University Clinic and Egas Moniz Dental Clinic. Inclusion criteria were age 18 to 50 years; a diagnosis of headache attributed to TMD according to the Diagnostic Criteria for Temporomandibular Disorders (DC-TMD);^14^and written informed consent. Exclusion criteria were absence of headache; presence of headache that were not attributed to TMD according to DC-TMD;^14^ the presence of any musculoskeletal, psychiatric, cardiovascular, pulmonary, metabolic, and/or neurological disease; other medical reasons that prevented the performance of moderate-intensity aerobic exercise; a history of facial trauma; use of orthodontic appliances; and use of painkillers and/or anti-inflammatory drugs in the 48 hours prior to data collection. Sample size was calculated using GPower 3.0, version 3.0.10 (Heinrich-Heine-Universität, Dusseldorf, Germany), and 45 subjects were recruited according to the highest value obtained in the different statistical methodologies used, with an alpha of 5% and a power of 80%.

The study protocol was approved by the Ethical Committee of the Egas Moniz University Institute on February 13, 2019 (reference number: 675). All individuals provided informed consent in accordance with the Helsinki Declaration and understood that they were free to withdraw from the study at any time.

The null hypothesis is “patients with headache attributed to TMD undergoing a combined program of therapeutic exercises with aerobic exercises (G2) will not significantly reduce the frequency, intensity and adverse impact of headaches”. The hypothesis one is “patients with headache attributed to TMD undergoing a combined program of therapeutic exercises with aerobic exercises (G2) will significantly reduce the frequency, intensity and adverse impact of headache”.

Patients were distributed in three exercise groups. G1 to who did not agree to perform aerobic exercise, and G2 and G3, randomly, to the remaining patients. G1 carried out a therapeutic exercise protocol for the masticatory muscles, G2 carried out the same protocol accompanied by an aerobic exercise program, and G3 had the same aerobic exercise program as G2. At the first assessment (A01), data on the frequency, intensity, and impact of headaches were collected. Two weeks later, a new evaluation (A02) was performed before the start of the interventions. Afterward, the patients in G1, G2, and G3 started their respective exercise programs. Eight weeks later, a new evaluation (A1) was performed 48h after the end of the exercise programs. Eight to twelve weeks after ending the intervention, patients returned to the hospital for the last evaluation (A2). All evaluation data were collected by an independent researcher blinded to group allocation.

Patients in the G1 group participated in a weekly exercise session for a period of 8 weeks. The physiotherapy session was the same for all patients; the techniques were always applied in the same sequence and performed by the same physiotherapist for 30 minutes. Each session consisted of the following techniques: bilateral compression with a transverse and longitudinal massage of the masseter muscle, a bilateral longitudinal massage of the temporal muscle, a bilateral compression of the medial pterygoid muscle, a bilateral passive stretching of the masseter and medial pterygoid, isotonic strengthening exercises through a resisted mouth opening and closing and a resisted left and right deviation (10 repetitions of each exercise), and coordination exercises through mouth opening and closing exercises and laterals (10 repetitions of each exercise).^15-17^

Patients in the G2 group participated in a weekly physiotherapy session, the same described for G1, for 8 weeks. The G2 aerobic exercise program included two weekly cycle ergometer training sessions, which were always supervised by the same physiotherapist and lasted for 30 min. Patients cycled for the first 5 min (warm-up period) with an intensity of 50% of the heart rate reserve (HRR), the next 24 minutes with an intensity of 70% of HRR, and the last minute at 50% of HRR for active recovery. The speed and/or resistance of the cycle ergometer was adjusted throughout the training period to maintain the exercise intensity within a predefined value. HRR was determined according to the Karvonen formula^18^ and the resting heart rate (HR) was assessed on 3 consecutive days after 5 min of rest in a chair with the arms supported. The average value was calculated and used as the resting HR.

Patients in G3 underwent 2 cycle ergometer training sessions per week for 8 weeks, which were always supervised by the same physiotherapist and lasted 30 min. The protocol was the same as that defined for G2.

Headache frequency/intensity were assessed through entries to a headache diary during the period between the first and the last evaluation. Patients were directed to record all episodes of headache experienced weekly and determine the intensity of the headache using the numeric pain rating scale (NPRS) of 11 items. The time elapsed since the first episode of headache was assessed through the DC-TMD symptom questionnaire, and the mapping through self-reported Headache Impact Test (HIT-6) questionnaire.^19^

Patients who had headache attributed to TMD at the end of the intervention period were referred to a multidisciplinary medical team specializing in headache.

## Statistical analysis

Categorical variables were analyzed using Pearson’s chi-square test to confirm equality between groups at A01. Whereas for continuous variables, a non-parametric Kruskal-Wallis test was performed to confirm equality between groups at A01. A two-way mixed ANOVA was used to assess the differences between variables at the various assessment times (random factor) and for the 3 intervention groups (fixed factor). A 95% confidence interval (CI) was determined for all tests, and a 5% significance level was used. The SPSS® version 26 (IBM Corp., Armonk, NY) was used for all statistical analyses. Cohen’s d effect size was also calculated; effect sizes up to 0.2 were considered irrelevant, those between 0.2 and 0.5 were considered small, those between 0.5 and 0.8 were considered moderate, and values above 0.8 were considered large.

## Results

The sample consisted of 52 patients; however, we excluded those without headaches attributed to TMD at the beginning of the study and studied the evolution between groups. We were left with 36 patients, 12 per group, still with no differences between groups at A0 (χ2=1.83, p=0.4). Each group was composed of 10 women and 2 men ranging from 18 to 35 years of age. The mean age and standard deviation were 26.3±٥.7 years for G1, 26.0±4.6 years for G2, and 25.8±2.94 years for G3. All patients in the 3 groups reported the presence of headaches attributed to TMD mostly for more than 6 months (χ^2^=3.45, p=0.71).

### Headache frequency and intensity

The results showed that there was an association between the intervention programs (A1) and the decrease in the number of patients who reported the presence of headache. In G1, only 2 patients (16.7%) reported headache at A1; in G2, none of the patients reported episodes of headache at A1; and in G3, 10 patients reported headache at A1 (83.3%). At A2, there was only one patient in G1 and G3 reporting headache again.

**Figure 1:**
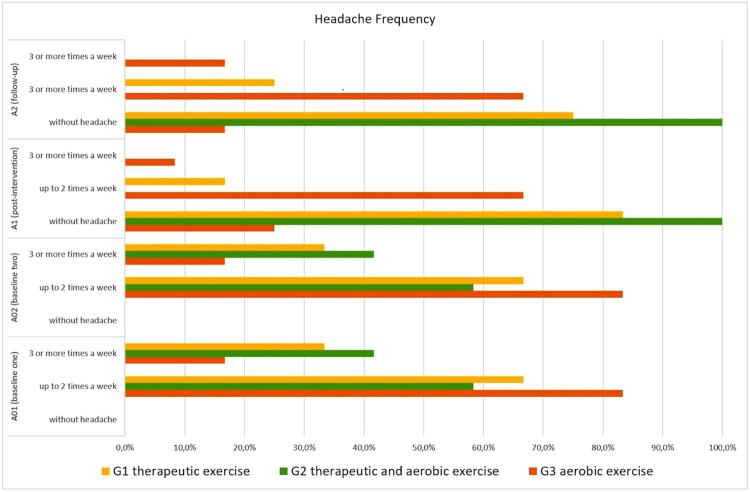
Changes in headache episodes per week assessed at different time points according to intervention

Regarding the number of headache episodes per week, there was no difference between the 3 groups (χ^2^=1.83, p=0.4) at A01 and A02. There was a decrease in the number of episodes per week after the 8-week intervention period. At A1 in G1, only 16.7% of patients (n=2) reported up to 2 episodes of headache per week, whereas in G2, there was no headache episodes; in G3, 66.7% of patients reported up to two episodes of headache per week, and 8.3%% reported three or more. Between A1 and A2, there was a slight increase in patients who reported headaches attributed to TMD up to two episodes per week in G1 and those who reported up to two episodes per week and three or more episodes in G3. In G2, all patients continued reporting having no headache episodes (Figure 1).

**Figure 2:**
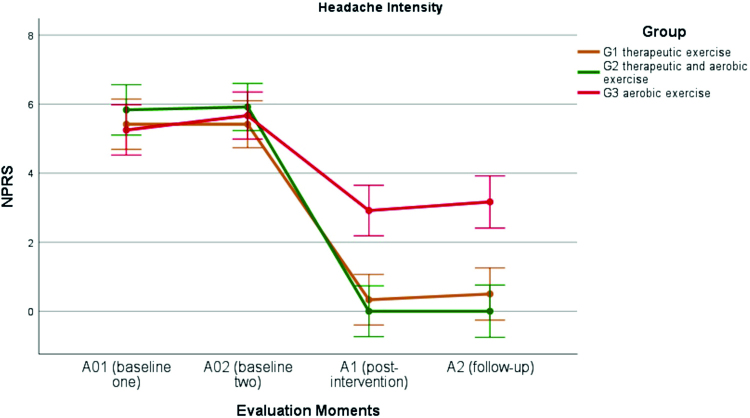
Changes in headache intensity at the different times points (NPRS – numeric pain rating scale)

The intensity of headache in all groups (according to the NPRS) was not significantly different at A01 and A02 (H=0.65, p=0.53). After conducting the intervention programs, there was a significant drop in the average headache intensity (F [707.5, 3]=311.9, p<0.001), which was more drastic in G2, where the NPRS average of 5.8 went to 0. In G1, the NPRS average went from 5.4 to 0.3, whereas in G3, the NPRS average went from 5.2 to 2.9 (Figure 2). Between A1 and A2, in G1 (NPRS of 0.5 [95% CI: -0.256, 1.256]) and G3 (NPRS of 3.2 [95% CI: 2.411, 3.923]), there tended to be an increase in headache intensity (according to NPRS), but it was not significant, nor did it have a notable effect size. G2 showed no changes in A2. Cohen’s d analysis showed a large effect in G2 (d=2.5) and G1 (d=2.3), and a moderate effect in G3 (d=0.8).

### Headache impact test

Analysis of the three groups at A01 and A02 verified that the HIT-6 was not significantly different (H=0.25, p=0.78). Changes in HIT-6 after the intervention programs (A1) were favorable for patients in G1 and G2, being significantly different from G3. Between A1 and A2, there was a slight increase in HIT-6, but it was not significant, nor did it have a notable effect size (Table 1). Finally, the Cohen’s analysis showed a large effect in G2 and G1 with values of d=2.3 and 3.2, respectively, and a small effect of 0.4 in G3.

**Table 1:** Changes in the headache impact test score (HIT-6) at different time points

	G1 (mean)	Lower Bound	Upper Bound	G2 (mean)	Lower Bound	Upper Bound	G3 (mean)	Lower Bound	Upper Bound
**HIT-6**		**95% Confidence Interval**			**95% Confidence Interval**			**95% Confidence Interval**	

A01	66.9	56.719	77.148	62.9	52.719	73.148	62.2	51.985	72.415
A02	67.6	57.642	77.558	63.7	53.709	73.624	62.5	52.576	72.491
A1	50.7	38.008	63.459	49.5	36.808	62.259	59.3	46.541	71.992
A2	55.1	42.998	67.268	51.7	39.532	63.802	59.8	47.665	71.935

Subtitle: G1 – experimental group that carried out a therapeutic exercise program; G2 – experimental group that associated a therapeutic exercise program with aerobic exercise; G3 – experimental group that carried out a therapeutic exercise program; A01– evaluation baseline one; A02 – evaluation baseline two; A1 – evaluation post-intervention three; A2 – follow-up; HIT-6 – Headache Impact Test (36–78 points).

## Discussion

To our knowledge, this study is the first to evaluate the effects of a combined program of therapeutic exercises and aerobic exercises on headaches attributed to TMD. The null hypothesis was rejected, because combined program of therapeutic exercises with aerobic exercises (G2) significantly reduce the frequency, intensity and adverse impact of headaches attributed to TMD.

Headaches and TMD are comorbid conditions, that is, they occur frequently in the same individual.^1,2,7,14,20-22^ Regarding the time elapsed since the onset of symptoms, only 2 patients (16.7%) from G1 and G2 reported the presence of pain for less than 6 months, whereas only one in each group reported the presence of a headache for less than 3 months. According to the definition of chronic pain,^23,24^ this seems to indicate that most patients had a chronic headache if defined as lasting a period of 3 months from the first episode. Chronic pain is maintained in part by central sensitization, a phenomenon seems to be associated by prolonged, long-lasting nociceptive stimulation or by decreased pain inhibition. Changes in these circuits may change perception of pain independent of peripheral neural activation in patients.^25^ The fact that most of patients that participated in the study reported headache attributed to TMD for more than 6 months may indicate that they have central sensitization.

The number of headache attributed to TMD episodes reported after the intervention programs decreased in all groups, and in G2, none of the patients reported headaches at A1. In G1, only two patients (16.7%) reported a headache, with a maximum frequency of 2 episodes per week, and in G3, only 2 patients (16.7%) reported having no headaches. After the intervention in G2, G1, and G3, the intensity of headaches attributed to TMD significantly decreased, despite the greater difference in the first two groups compared to the third, also in G2 all patients stopped reporting pain. The results obtained in G1 are in line with those observed by Mesa-Jiménez, et al.^26^ (2015) which a decrease in the frequency and intensity of headaches was identified after performing a therapeutic exercise protocol. However, in the same study, a manual therapy program was compared with a pharmacological approach, which makes the comparison of results more difficult.^26^ The programs with identified therapeutic exercises are quite heterogeneous but have been shown to be effective in reducing pain in various health conditions.^27-29^ However, the results obtained in our study seem to indicate that the therapeutic exercise program developed for the masticatory muscles (type and sequence of exercises, number of repetitions, weekly frequency and time period of eight weeks) led to a significant reduction in the intensity and frequency of headaches attributed to TMD and a retention of these effects in the short / medium term.

In G3, the results were also in line with the literature. A systematic review conducted by Machado-Oliveira, et al.^13^ (2020) observed that aerobic exercise was effective in reducing the intensity of headaches by 75%, a higher value than that obtained in our study, in which the reduction was approximately 50%. In the systematic review, the 25% reduction of frequency was effective, and the same value that was found in our study. In contrast, Lemmens, et al.^30^ (2019) reported a moderate decrease in headache intensity between 20-54%, a value that is in-line with what was observed in this study. Despite these results, the heterogeneity of aerobic exercise protocols in the literature is marked. Therefore, it is not possible to compare the effects of the aerobic exercise defined in our study and those performed in other investigations.

In our study, the intervention program that associated therapeutic exercises with aerobic exercises (G2) showed the best results, which suggests that both types of intervention enhance the results obtained, possibly due to the association of the effects of therapeutic exercises with the effects of aerobic exercise. This is namely due to vasomotor changes and vasovagal activity; the release of endocannabinoids, endorphins, neurotrophic factors, and anti-inflammatory factors; and the improvement of hormonal regulation and neurotransmitter function resulting from aerobic exercise.^31^

The adverse impact of headaches on patient lives is multidimensional.^32^ Therefore, it is important to analyze effects on different aspects of daily living. The results of HIT-6 showed a decrease in G1 and G2 scores after completion of the intervention programs. The minimum clinically important difference defined for HIT-6 was between 2.5 and 5.5 for migraines^33^ and 8 points for tension-type headache (TTH),^34^ with no studies identified for headaches attributed to TMD. Compared to previous studies, the present G1/G2 intervention programs led to important clinical improvement in the impact of headaches.

In G3, the difference between the evaluations was 4.5 points, which, according to the studies identified, may or may not be indicative of an improvement in the impact of headache in this group. However, according to the HIT-6 cut-offs, the patients in G3 stopped having a severe headache impact and started to have a moderate impact.

The G1 HIT-6 score results are in-line with those verified in other studies, although the differences obtained in our study were greater than those identified previously.^35,36^ The result of the applied therapeutic exercise program (G1) was in line with other studies, but with differences in the HIT-6 with greater magnitude of effects. Regarding G3, no studies that used HIT-6 to assess the adverse impact of headaches on patients have been identified, therefore, impeding this comparative analysis.

In our study, the intervention with only therapeutic exercises (G1) showed best HIT-6 results. This group also reported a reduction in the frequency and intensity of headaches, which would have led to a reduction in the adverse impact of headaches. However, G2 was expected to achieve the greatest reduction in the adverse impact of headache, since it has the least limitation in its activities compared to other groups, and the patients reported fewer episodes of headache and less pain. In fact, in terms of the HIT-6 average score, the lowest value was obtained by G2, although this was not significantly different from G1. The perception of the impact of headaches on activities of daily living depend on a set of factors including self-efficacy strategies, perceived risk of persistent pain, feeling of well-being, individual strategies to deal with pain and resilience. The referred multidimensionality may contribute to the frequency and intensity of similar headaches being perceived in different ways by different individuals, with some identifying greater limitations in their activities and attributing a greater dimension to headaches.

In the last follow-up (A2), HIT-6 scores increased slightly, which was not significant according to the minimum clinically important difference values reported for the impact of headache. That is, 8-12 weeks after the end of the intervention programs, the results were maintained, which is an indicator that they allow to retain the effects obtained. However, the upward trend in HIT-6 values may indicate that in the future it will be indicated to keep monitoring patients in terms of extended timeline as an increase of number to see if this trend continues or stabilizes. Regarding impact, any of the groups still had the same results as those that were observed after the end of the intervention programs, therefore, it is possible to say that overall, the positive effects were maintained.

This study has some limitations, such as the small, convenience-based sample, which means that the findings may not be generalizable to other populations. The sample also included only subjects aged between 18 and 35 years, which does not allow the extrapolation of results to other age groups. The non-acceptance rate by several patients to join G2 and G3, which includes the performance of the aerobic exercise program, may have contributed to some heterogeneity between groups at baseline, and the diagnosis of headache based only on the DC-TMD may have led to some failures.

Future studies should recruit a larger sample and randomize patients across the three groups so that the results can be extrapolated to other populations and the type of headaches can be categorized not only based on DC-TMD, but also with the collaboration of a neurologist. Chronicity of pain should be assessed using the Graded Chronic Pain Scale and not just the time since the first episode of headaches. An intervention program that includes aerobic exercises of moderate-intensity to facilitate the patients’ adherence and conducting an intervention program with a shorter duration (6 weeks) should also be considered to check if similar results could be obtained in a shorter period.

## Conclusion

The greatest effects on decreasing headache attributed to TMD frequency and intensity were observed in the group whose intervention program consisted of therapeutic and aerobic exercises (G2), in which none of the patients reported headaches attributed to TMD at A1. Although the differences were not significant considering the group with the therapeutic exercise program (G1), in which only two patients reported having a headache attributed to TMD. There were no significant differences in follow-up (A2) compared to A1. In conclusion, programs that associated therapeutic with aerobic exercises had positive effects in patients with headache attributed to TMD after an eight-week intervention period and at the end of interventions, meaning that these programs maintained a positive effect in the short/medium term.
